# Characterization of auditory synaptic inputs to gerbil perirhinal cortex

**DOI:** 10.3389/fncir.2015.00040

**Published:** 2015-08-14

**Authors:** Vibhakar C. Kotak, Todd M. Mowery, Dan H. Sanes

**Affiliations:** Center for Neural Science, New York UniversityNew York, NY, USA

**Keywords:** synaptic, intrinsic, calcium, auditory cortex, medial geniculate

## Abstract

The representation of acoustic cues involves regions downstream from the auditory cortex (ACx). One such area, the perirhinal cortex (PRh), processes sensory signals containing mnemonic information. Therefore, our goal was to assess whether PRh receives auditory inputs from the auditory thalamus (MG) and ACx in an auditory thalamocortical brain slice preparation and characterize these afferent-driven synaptic properties. When the MG or ACx was electrically stimulated, synaptic responses were recorded from the PRh neurons. Blockade of type A gamma-aminobutyric acid (GABA-A) receptors dramatically increased the amplitude of evoked excitatory potentials. Stimulation of the MG or ACx also evoked calcium transients in most PRh neurons. Separately, when fluoro ruby was injected in ACx *in vivo*, anterogradely labeled axons and terminals were observed in the PRh. Collectively, these data show that the PRh integrates auditory information from the MG and ACx and that auditory driven inhibition dominates the postsynaptic responses in a non-sensory cortical region downstream from the ACx.

## Introduction

The representation of auditory experience involves downstream processing beyond the auditory cortex (ACx). One region that processes sensory cues and is thought to represent mnemonic information is the perirhinal cortex (PRh; Suzuki and Naya, 2014). In rodents, the PRh is located in close proximity to ACx, and mediates the flow of information in and out of hippocampal regions associated with spatial and recognition memory (reviews: Sacchetti et al., 1999; Munoz-Lopez et al., 2010; Kealy and Commins, 2011; Saldeitis et al., 2014). Furthermore, PRh lesions disrupt behaviors that involve auditory cues (Corodimas and LeDoux, 1995; Kholodar-Smith et al., 2008a,b; Bang and Brown, 2009a, b; Gastelum et al., 2012). These observations motivated us to characterize the synaptic drive to the PRh from auditory regions in previously developed thalamocortical brain slice preparation (Cruikshank et al., 2002; Kotak et al., 2005).

The rodent PRh is connected with both subcortical and cortical structures. Injection of tracer dyes into the PRh reveals projections from areas that convey auditory information, including the MG, ACx, association cortices, and the amygdala (Krettek and Price, 1974; Deacon et al., 1983; McDonald and Jackson, 1987; Burwell and Amaral, 1998; Doron and Ledoux, 1999; Pikkarainen and Pitkänen, 2001; Kimura et al., 2003; Furtak et al., 2007b). In fact, PRh neurons are responsive to sound, including vocalizations (Allen et al., 2007; Sadananda et al., 2008). Furthermore, long-latency, auditory-evoked potentials recorded in motor cortex *in vivo* are abolished when PRh is lesioned (Kyuhou et al., 2003). Here, we used a peri-horizontal brain slice preparation known to preserve functional connections between the MG and ACx (Cruikshank et al., 2002; Kotak et al., 2005). Injection of fluoro ruby in the ACx *in vivo* confirmed that anterograde labeled terminals were observed within the PRh. We then demonstrated that these slices preserve functional connections from both the MG and ACx to the PRh, thus allowing us to characterize auditory afferent-driven synaptic and calcium responses.

## Materials and Methods

### Brain Slice Preparation

**Figure 1:**
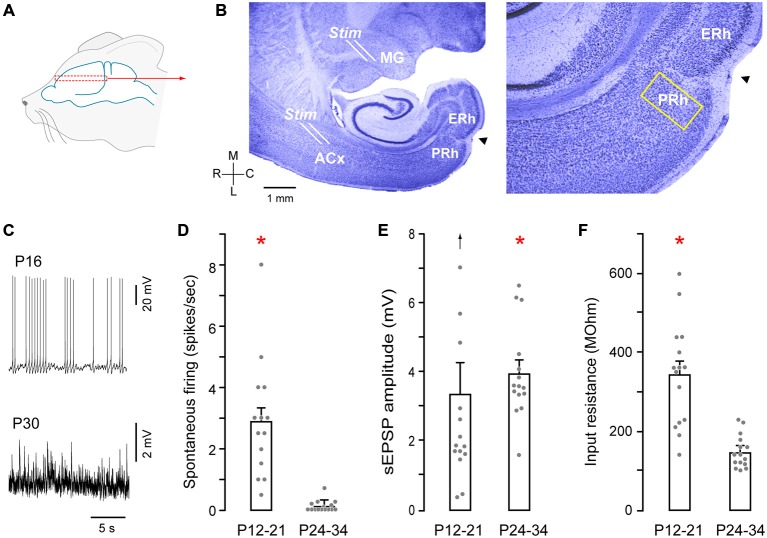
**Auditory thalamocortical—perirhinal cortex (PRh) brain slice preparation. (A)** Schematic of approximate region from which brain slices were sectioned (dashed rectangle). **(B)** Nissl stained perihorizontal sections showing major landmarks (left). These include the auditory thalamus (MG), auditory cortex (ACx), PRh, entorhinal cortex (ERh) and the rhinal fissure (arrowhead). The yellow rectangle at the PRh represents an approximately 200 μm area within which recordings were made. About 15% of these recordings were from cells located within 50 μm of rostral and medial to the fissure (arrowhead). Afferents emerging out of the MG were stimulated by a stimulating electrode placed at the rostral border of the MG (see **B**, Stim). ACx was stimulated by another stimulating electrode at the sites shown (Stim). High magnification micrograph (right) shows the architecture of PRh laminae and the rhinal fissure (arrowhead). **(C)** Representative traces show spontaneous spiking (P16) and synaptic (P30) activity in PRh. **(D)** Bar graph shows the significantly (red asterisk) higher discharge in P12–21 neurons (mean ± SEM; filled symbols represent individual neurons). **(E)** Bar graph shows a small, but significant (red asterisk) age difference for stimulation evoked a slow excitatory postsynaptic potential (sEPSP) amplitudes (upward arrow indicates one outlier). **(F)** Bar graph shows significantly (red asterisk) higher input resistance in P12–21 neurons.

Gerbils (*Meriones unguiculatus*), aged postnatal days (P) 12–34 were obtained from breeding pairs (Charles River). All protocols were approved by the Institutional Animal Care and Use Committees at New York University. Animals were first anesthetized by an intraperitoneal injection of 5% chloral hydrate (500 mg/kg body weight, Sigma) made as an aqueous solution. Brains were then dissected in 0–4°C oxygenated artificial cerebrospinal fluid (ACSF, in mM: 123 Nacl, 4 KCl, 1.2 KH_2_PO_4_, 1.3 MgSO_4_, 24 NaHCO_3_, 15 glucose, 2.4 CaCl_2_, 0.2 ascorbic acid; pH 7.3 after bubbling with 95% O2/5% CO_2_). The brain was vibratome-sectioned at 400 μm peri-horizontally at a 15–25° (depending on age) lateral-to-medial angle as described previously (Cruikshank et al., 2002; Kotak et al., 2005). Briefly, brains were mounted ventral side up at an angle (15° angle for P12–21 brains; 25° for P24–34 brains) on a TPI vibratome. An approximately 1.5 mm section was first cut to access the thalamocortical pathway, and 2–3 slices were then obtained containing the PRh (Figure 1). Thus, the rostro-caudal extent of the rhinal fissure was within the first 1.5 mm of tissue, and we did not record from this area. Pyramidal neurons located within layers (L) 2–5 were identified under IR-DIC optics and patched for recordings. The recorded PRh neurons were within 200 μm rostral and medial to the rhinal fissure (Burwell et al., 1995; Figures 1A,B). To validate that the recorded neurons were healthy, we monitored the resting membrane potential, action potentials elicited by depolarizing current (10 pA steps, 1500 ms pulses until firing threshold, 0.05 Hz). Hyperpolarizing current pulses were also delivered up to −100 pA.

To validate that functional connections existed between MG and ACx, a bipolar stimulating electrode (matrix electrode; exposed tips 150 μm apart; FHC, ME) was placed at the rostral border of the MG, and stimulation at this site evoked field responses in layer 5/6 of ACx. We did not attempt to characterize MG-evoked responses in the PRh by stimulating different sub-divisions of the MG. A second stimulating electrode was then placed on ACx L5/6 (Figure 1B) to ensure that thalamorecipient ACx could be independently stimulated to evoke responses in the PRh. It is possible that multiple cell types within L5/6 may have been activated and that synaptic activation of the supragranular layers also occurred. Thereafter, synaptic responses were recorded in PRh following stimulation of either MG or ACx (1–5 ms; 10 mA). The stimulus duration and intensity were employed to maximize activating long-range synaptic inputs onto PRh neurons, and we did not observe any local injury following stimulation. Generally, MG and ACx-evoked synaptic responses were obtained from the same brain slices and PRh neurons.

### Recordings and Pharmacological Manipulations

Whole-cell recordings were first obtained in current-clamp mode, described previously (Mowery et al., 2015), using the Warner’s patch-clamp amplifier PC-501A. Briefly, the internal solution contained (in mM): 130 potassium gluconate, 5 KCl, 2 MgCl_2_, 2 ATP, 0.3 GTP, 0.6 EGTA, 10 Hepes (pH = 7.2). To isolate excitatory responses, (−)-bicuculline methbromide, 1S, 9R (BIC, 20 μm, Sigma) was added to the ACSF to block type A gamma-aminobutyric acid (GABA_A_) receptor-mediated inhibition. BIC treatment also revealed whether GABA_A_ receptor-mediated shunting inhibition contributed to the magnitude of evoked excitatory postsynaptic potentials (EPSPs). Cells with resting membrane potential of −50 mV or less and overshooting action potentials were included. A second set of recordings was obtained in voltage-clamp mode using the same PC-501A amplifier, for which potassium gluconate was replaced by equimolar Cs-gluconate (Sigma) to block potassium channels, and QX-314 ([2(triethylamino)-N-(2,6-dimethyl-phenyl) acetamine], 5 mM, Alamone, Israel) was added to block sodium channels. Under these recording conditions, series resistance varied between 10 and 40 MΩ and this was compensated by approximately 70%. Neurons with series resistance exceeding 40 MΩ were not included in the analysis. Liquid junction potentials were not corrected.

The data were digitized at 10 kHz using an iMAC running custom Igor-based software (WaveMetrics, v4.04) and analyzed offline in a manner similar to that described previously (Takesian et al., 2012). Statistical tests (Distribution and Goodness of Fit, ANOVA followed by students’ *t*-test, or Wilcoxon *X*^2^ test) were performed using the SAS-based JMP 9.0.1 package. For non-parametric unpaired data from different ages, a Wilcoxon Mann-Whitney rank sum test was used.

### Calcium Imaging

To further validate functional connectivity from the MG or ACX to PRh, brain slices (400 μm) were bulk loaded with fluo-4, AM dissolved in 20% (w/v) pluronic acid F-127 (Invitrogen) at 50 μg of fluo-4, AM in 48 μL of DMSO and 2 μL of pluronic acid for a final concentration of 1 mM (Yuste et al., 2011). The concentration of the dye in the chamber was 10–20 μm. Slices were bulk loaded for ~30 min at 32°C in a dark and washed in ACSF. Imaging and acquisition was performed using a Hamamatsu Flash 2.8 camera on an Olympus microscope, and processed with HC Image software. Images were acquired at 488 nm excitation wavelength with a frame rate of 50–100 Hz. A triggered event marker was used to time the onset of MG or ACx stimulation. For offline analyses, individual PRh neurons were selected as regions of interest (ROIs). Calcium imaging was not performed in conjunction with whole cell recordings, or following any pharmacological manipulations. Thus, the aim of imaging was to support the synaptic connectivity data and not to use to assess voltage- or ligand-gated mechanisms.

### Neuroanatomical Tracing

To determine whether anatomical evidence could support functional connections established by evoked synaptic and calcium responses, four male gerbils were injected with tracers to demonstrate connectivity between ACx and PRh (Figure 4). Animals were anesthetized (chloral hydrate; 500 mg/kg body weight, i.p.), and a craniotomy was made 3 mm rostral and 6.5 mm lateral to lambda over ACx (Budinger et al., 2000). 100 nl of anterograde tracer (10% fluoro ruby) was injected at 3 different depths; 1.2, 0.8, and 0.3 mm below the pial surface with a Nanoject II injector (Drummond Scientific). The injector was then retracted, the craniotomy site was covered with sterile bone wax and the surgical opening closed with Vetbond. After 1 week of survival, animals were anesthetized, and perfused with 4% paraformaldehyde. The brains were vibratome sectioned (Leica) at 150 μm at the same plane used for the *in vitro* brain slice preparation described above. Sections were mounted on gelatin-coated slides, cleared with xylene, cover-slipped with VECTASHIELD mounting Medium, and images were captured (Zeiss AxioCam ax10; QImaging).

## Results

### PRh Properties in Brain Slices

Recordings were made from PRh neurons in a perihorizontal brain slice preparation, (Figures 1A,B). We first analyzed membrane and firing properties of PRh neurons as a function of age, pooling the data into two groups: P12–21 and P24–34 (Figures 1C–F). There was a significantly higher spontaneous action potential rate in P12–21 neurons, as compared to P24–34 (P12–21: 2.9 ± 0.48 Hz, *n* = 15 vs. P24–34: 0.1 ± 0.4 Hz, *n* = 15; Shapiro-Wilk: *W* = 0.77; *p* = 0.0001, *n* = 30; Wilcoxon Mann Whitney rank sum test, *X*^2^ = 22.2, *p* = 0.0001). In addition, the mean stimulation evoked a slow excitatory postsynaptic potential (sEPSP) amplitude was greater in P24–34 neurons (P12–21: 3.3 ± 0.9 Hz, *n* = 15 vs. P24–34: 3.9 ± 0.4 Hz, *n* = 15; Shapiro-Wilk: *W* = 0.81, *p* = 0.0001, *n* = 30; Mann Whitney rank sum test, *X*^2^ = 4.22, *p* = 0.04). However, there was no significant difference sEPSP frequency between the two age groups (P12–21: 2.6 ± 0.4 Hz, *n* = 15 vs. P24–34: 2.5 ± 0.2 Hz, *n* = 15; Wilcoxon Mann Whitney rank sum test, *X*^2^ = 0.17 *p* = 0.67). Finally, input resistance was significantly greater at P12–21 (P12–21: 331 ± 34 MΩ, *n* = 15 vs. P24–34: 148.3 ± 10 MΩ, *n* = 15; Shapiro-Wilk: *W* = 0.82; *p* = 0.001, *n* = 30; Wilcoxon Mann Whitney rank sum test, *X*^2^ = 16.75, *p* = 0.0001).

### MG- and ACx-Evoked Responses in PRh

**Figure 2:**
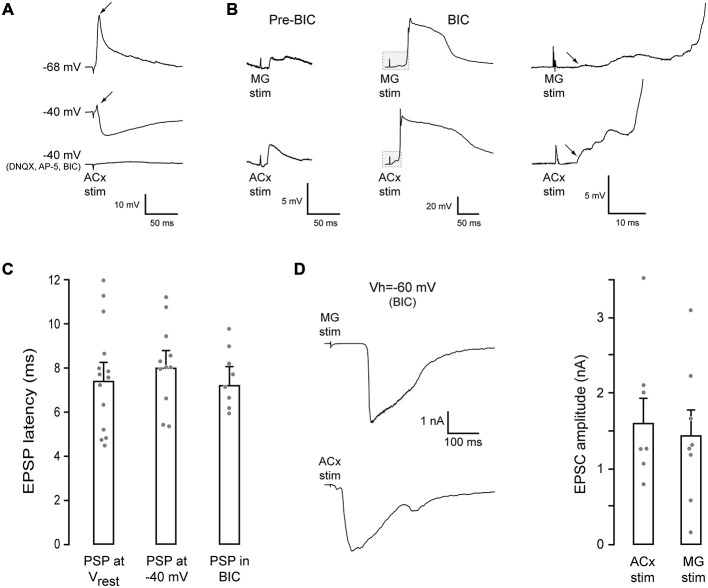
**MG- and ACx-evoked synaptic responses in PRh. (A)** Example traces of ACx-evoked synaptic responses in a PRh neuron. At rest (top trace, −68 mV), the response was dominated by an EPSP (arrow). When the membrane potential was held at −40 mV (middle trace), an IPSP was revealed, but a small EPSP remained (arrow). Blockade of glutamate and GABA_A_ receptors abolished the synaptic response (bottom trace). **(B)** MG (top) or ACx (bottom) stimulation evoked PSPs in another PRh neurons (pre-BIC, resting potential = −64 mV). After bicuculline (BIC) exposure, the evoked excitatory postsynaptic potentials (EPSPs) were dramatically increased, evoking spikes. The initial portion of each trace (gray box) is shown at a high gain and temporal resolution (right) to illustrate the shortest latency EPSP (arrows). **(C)** Bar graph shows no difference in the shortest latency EPEPs for each condition. **(D)** Example MG- (top) and ACx-evoked (bottom) EPSCs in PRh neurons in the presence of BIC (left). Bar graph shows no difference between MG- and ACx-evoked EPSC amplitudes.

To characterize afferent connectivity to the PRh, whole-cell current and voltage-clamp recordings were obtained in a brain slice. Stimulation of either the MG or the ACx (as shown in Figure 1A) evoked mixed excitatory-inhibitory responses in PRh neurons. As shown in Figure 2A, excitatory potentials dominated at the neurons’ resting potential. In contrast, an inhibitory postsynaptic potential (IPSP) was revealed when the neuron was held at a depolarized membrane potential (−40 mV). Figure 2B shows both MG- and ACx-evoked PSPs in the absence of BIC (pre-BIC) and following of BIC exposure (BIC). The ACx-evoked IPSPs recorded in PRh neurons at a holding membrane potential of −40 mV were −4.6 ± 0.8 mV (*n* = 11). When slices were bathed with the selective AMPA receptor blocker, 6,7-dinitroquinoxaline-2,3-dione (DNQX, 20 μm), the NMDA receptor blocker, ((2*R*)-amino-5-phosphonovaleric acid; (2*R*)-amino-5-phosphonopentanoate; AP-5, 50 μm) and BIC (20 μm), the synaptic potentials were abolished. Separately, there was a robust effect of BIC on PSP amplitude (*n* = 8). Whereas control PSPs were 5.5 ± 1.1 mV, BIC exposure led to very large depolarizing PSPs and action potentials.

We next asked whether facilitation of EPSPs in the presence of BIC was restricted to PRh, or whether a similar phenomenon occurred in ACx (i.e., whether BIC treatment led to equivalently large PSPs in the ACx). A large increase in the depolarizing PSP amplitude and number of action potentials was also observed in the ACx L2/3 pyramidal neurons when L4/5 was stimulated in the presence of BIC (not shown).

Since the two stimulation sites, MG and ACx, were located at a relatively long distance from the PRh (Figure 1B), we measured the shortest latency synaptic response evoked by ACx stimulation (rise time of the first synaptic potential that was ≥0.5 mV). Latencies were obtained at each neuron’s resting membrane potential, when the membrane was held at −40 mV, and following exposure to BIC (Figure 2C). The latency was 7.6 ± 0.6 ms (*n* = 14) at the resting potential. Further, the PSP latency was unchanged by holding the membrane potential at −40 mV, or by BIC exposure (ANOVA: *F* = 0.31, *p* = 0.73, *n* = 33). The latency at a membrane potential of −40 mV was 8.2 ± 0.6 ms (*n* = 11), and the latency in the presence of BIC was 7.6 ± 0.5 ms (*n* = 8). Thus, the shortest excitatory latencies appear to precede inhibition (Figure 2B).

To determine the magnitude of MG- and ACx-evoked excitatory postsynaptic currents (EPSCs), voltage-clamp recordings were performed in the presence of BIC (V_HOLD_ = −60 mV). As shown in Figure 2D, the mean EPSC amplitudes did not differ significantly as a function of the stimulation site (MG-evoked: 1.4 ± 0.33 nA, *n* = 8 vs. ACx-evoked: 1.6 ± 0.32 nA, *n* = 8; *t* = 2.14, *p* = 0.7, *n* = 8).

### MG- and ACx-Evoked Calcium Transients in PRh

**Figure 3:**
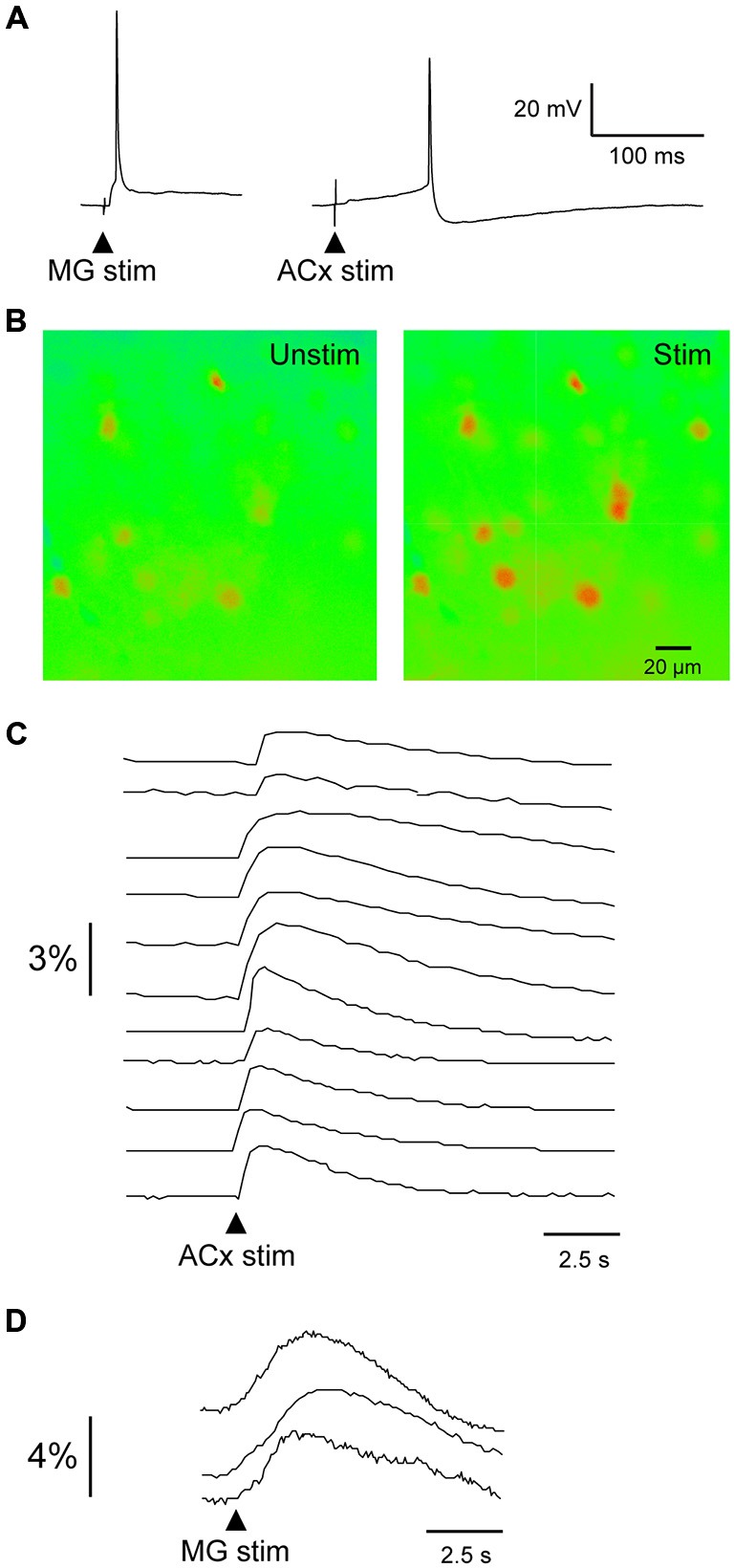
**Evoked calcium transients in PRh neurons. (A)** MG- (left) and ACx-evoked (right) action potentials recorded in PRh neurons from P19–20 slices. **(B)** Pseudo-colored PRh cells illustrate calcium fluorescence before and after a 1 ms stimulus pulse to ACx. **(C,D)** Relative intensity 14 PRh neurons that responded to stimulation of the ACx **(C)** or MG **(D)**. Arrowheads indicate stimulus onset.

To determine the extent of auditory connectivity to the PRh, we investigated whether PRh neurons responded to stimulation of MG or ACx. Since single electrical stimuli to MG or ACx in young brain slices evoked large EPSPs and action potentials (Figure 3A), we performed calcium imaging of PRh neurons from P11–18 slices (ACx stimulation: 120 PRh neurons; MG stimulation: 19 PRh neurons; *n* = 11 animals). Using an identical stimulation regimen to that employed for evoking synaptic responses, we observed a rise in fluo-4 fluorescence in about 70% of labeled neurons. Because fluo-4 is a non-ratiometric dye, imaging was performed at a single excitation wavelength (488 nm), and the data were analyzed for relative change in intensity and plotted as mean gray scale percentage against background fluorescence. Figure 3B shows pseudo-colored images of calcium fluorescence before and after ACx stimulation. Figures 3C,D show the temporal pattern of fluorescence in PRh neurons following ACx or MG stimulation. The percent change in mean gray scale fluorescence for all PRh neurons (*n* = 139 neurons) ranged from a minimum of 0.15% to a maximum of 3.9%. All calcium responses were obtained in the same region of the PRh from which whole-cell recordings were obtained.

### Anatomical Projections to PRh

**Figure 4:**
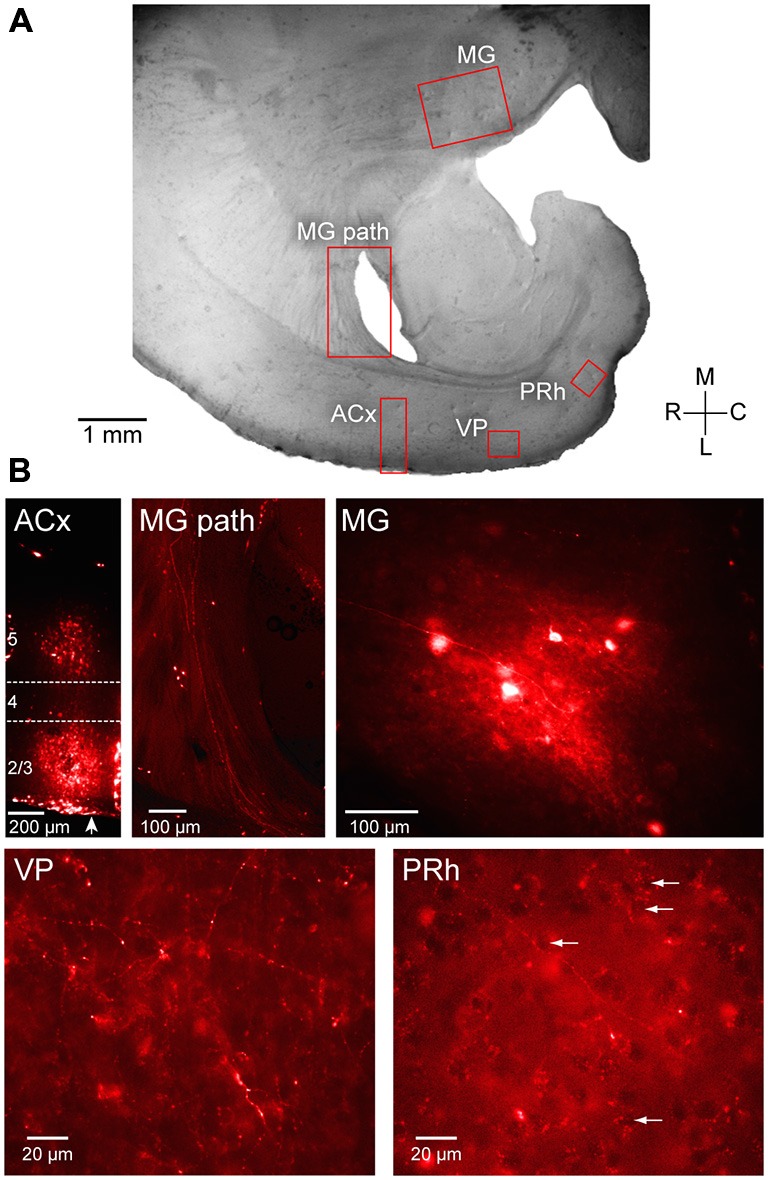
**Anatomical validation of projections from ACx to PRh.** Fluoro ruby was injected *in vivo* in to the ACx and labeling was examined after 1 week survival in peri-horizontal thalamocortical brain slices at a plane similar to freshly cut slices for physiology and imaging. **(A)** Horizontal brain slice showing the location of fluoro ruby injection in L2/3 and L5 and labeling (red boxes). **(B)** The individual fluorescent images show the ACx injection site (ACx), the retrogradely labeled MG thalamic fibers (MG pathway) and cell bodies (MG), and the anterogradely labeled fibers and terminals in the VP (VP) and the PRh. Several PRh cell bodies surrounded by labeled terminals are indicated by arrows. In the first left panel of **(B)**, an arrow indicates pial surface. L4 is shown located within the dashed lines while the injected dye appears diffusely spread within L2/3 and L5. All photomicrographs shown in **(B)** are along the same orientation as in panel **(A)**.

To examine auditory projections to PRh, fluoro ruby was pressure-injected *in vivo* into ACx (Figure 4B, left). Labeled fibers were subsequently examined in post-fixed sections taken at the same plane as the slice preparation used for electrophysiological recordings as shown in Figure 4A. To confirm that the injection site was in the ACx (Figure 4B), we observed that retrogradely labeled fibers and neurons were located within the MG (Figure 4B, middle and right). Anterogradely labeled fibers from ACx traveled to the ventro-posterior auditory cortex, VP (Figure 4B, bottom left). Furthermore, we observed labeled fibers and terminals within the PRh, indicating a direct projection from thalamorecipient ACx (Figure 4B, bottom right).

## Discussion

Our primary finding is that PRh, a key interface between sensory cortices and the hippocampus, is synaptically driven by both the auditory thalamus (MG) and cortex (ACx). The longest latency postsynaptic responses indicated that electrical stimulation of ACx or MG recruited polysynaptic pathways. However, we confirmed that direct projections from ACx to PRh are present in the plane of our brain slice preparation (Figure 4; Budinger et al., 2000). This projection may mediate putative monosynaptic, shorter latency responses recorded in PRh (Figure 2). A complete characterization of the functional pathway from MG to PRh will require a selective pharmacological blockade of putative interceding relays, such as VP, or selective optogenetic activation of MG.

Several auditory regions could plausibly contribute to the synaptic activation of PRh. Direct projections to PRh emerge from MG, the ACx, and rostral association cortices (Burwell and Amaral, 1998; Budinger et al., 2000; Kimura et al., 2003; Budinger and Scheich, 2009). Our electrophysiological, imaging, and anatomical results confirm and extend these observations. In addition, polysynaptic drive from primary ACx to PRh could be mediated by the ventro-posterior auditory cortex (VP), located between these two regions. For example, injection of biocytin into primary ACx or the adjacent auditory association cortices reveals anterogradely labeled axons and terminals in the VP (Budinger et al., 2000; Budinger and Scheich, 2009). Here, fluoro ruby injections into thalamorecipient ACx also revealed axons and nerve terminals in the VP (Figure 4E), implying that long latency responses in the PRh may be mediated via VP.

### Consequences of Auditory Inputs to PRh

The *in vivo* function of PRh has been most thoroughly studied with visual stimuli, and these studies suggest a contribution to mnemonic processing (Suzuki and Naya, 2014). Our findings support the notion that PRh also integrates auditory input for this purpose. In the rodent, PRh processes sound stimuli in conjunction with nociceptive stimuli for the purpose of generating fear responses (Rosen et al., 1992; Sacchetti et al., 1999; Lindquist et al., 2004; Bruchey and Gonzalez-Lima, 2006; Furtak et al., 2007a; Kholodar-Smith et al., 2008a,b; Bang and Brown, 2009a, b). For example, lesions of the PRh degrade fear conditioning associated with ultrasonic communication calls (Bang and Brown, 2009a). In this regard, it is noteworthy that behaviorally aversive auditory communication calls elicit significant discharge from basolateral amygdyla neurons that may provide auditory input to PRh (Grimsley et al., 2013).

Our findings indicate that the activation of projections from the MG or ACx elicit small EPSPs in the presence of feed-forward inhibition (Figure 2). Importantly, there is a very strong influence of development on PRh properties, which implies that future studies on mature plasticity should explore ages older than P35 (Figure 1). However, when GABAergic inhibition is blocked, the evoked EPSPs could elicit action potentials. Therefore, if only MG or ACx is activated, the feed-forward inhibition may diminish the discharge of PRh neurons. This is consistent with the model put forward by Unal et al. (2012, 2013), predicting that co-activation of more than one long-range excitatory projection is necessary to shift the balance within PRh in favor of excitation.

It has been shown that pharmacological enhancement of inhibitory function with a benzodiazepine (lorazepam) in the PRh can suppress long-term synaptic plasticity *in vitro* and diminish performance on a recognition memory task *in vivo* (Wan et al., 2004). Our finding that GABA_A_ receptor blockade dramatically facilitates EPSP amplitude is consistent with this observation (Figure 2B), and suggests that a disinhibitory mechanism could participate in synaptic plasticity, as observed in the ACx during learning (Sarro et al., 2015). Furthermore, when PRh brain slices are obtained from rats that had participated in a visual recognition-learning task, the neurons displayed a reduction in long-term excitatory synaptic long-term depression (Massey et al., 2008). Consistent with this observation, a synaptically evoked increase in calcium within PRh neurons may mediate calcium-dependent synaptic plasticity, especially during periods when the efficacy of inhibition decreases (Figures 2, 3). The functional implication of these findings is that the PRh may operate in conjunction with sensory cortices, including the ACx, during the acquisition and retrieval of memory (Squire and Zola-Morgan, 1991; Suzuki and Naya, 2014). In fact, recent findings suggest that PRh lesions can interfere with learning on task in which a visual feature determines whether target auditory stimuli are rewarded (Gastelum et al., 2012). Thus, our experimental preparation containing auditory forebrain pathways to the PRh, provides an opportunity to examine the effects of *in vivo* auditory manipulations on synaptic plasticity downstream of ACx.

## Author Contributions

VCK and TMM have contributed equally to the experiments and analysis. All authors conceptualized the experiments and wrote the manuscript.

## Conflict of Interest Statement

The authors declare that the research was conducted in the absence of any commercial or financial relationships that could be construed as a potential conflict of interest.
